# To the heart of the problem. mIGF-1: local effort for global impact

**DOI:** 10.18632/aging.100466

**Published:** 2012-06-23

**Authors:** Antonio Musarò

**Affiliations:** ^1^ Institute Pasteur Cenci-Bolognetti, DAHFMO-Unit of Histology and Medical Embryology, IIM, Sapienza University of Rome, Italy; ^2^ Edith Cowan University, Perth, Western Australia

The mammalian heart must maintain its structural and functional integrity for decades, yet the response to damage in this vital organ is remarkably inadequate and often results in heart failure. Moreover, patients with chronic heart failure show profound metabolic changes, leading to peripheral abnormalities in addition to an initial cardiac impairment. Several evidences have suggested a relationship between the IGF-1 system and cardiovascular disease. Many cardiovascular risk factors, such as sedentary lifestyle, diabetes, smoking, oxidized low-density lipoprotein, obesity, psychological distress and reduced coronary flow reserve, have been associated with reduced IGF-1 levels [1]. Conversely, human studies indicate that increased levels of IGF-1 are characterized by a decreased incidence of heart failure and mortality in elderly individuals [2]. Nevertheless, the fact that IGF-1 can act either as a circulating hormone or as a local growth factor has confounded previous analyses of animal models in which transgenic IGF synthesized in extra-hepatic tissues was released into the circulation. Locally acting mIGF-1 isoform improves muscle regeneration and counters muscle wasting associated with diseases, including sarcopenia, muscular dystrophy and ALS [3]. By contrast, circulating IGF-1 isoforms have been implicated in the restriction of lifespan and have contrasting effects on the heart when expressed as transgenes, variously promoting cell survival, or inducing prolonged hypertrophy with pathological consequences [4]. Another important mediator of cell homeostasis with cardio-protective effects is SIRT1, the largest and best characterized member of the Sirtuin family. Is there any functional interplay between IGF-1 signaling and SIRT1 expression and activity? The study of Bolasco *et al* [5] addresses this point and offers some fascinating insights into the molecular mechanism by which the cardiac mIGF-1/SIRT1 pathway in mice, has a global impact on the regulation of the immune system, the arterial blood pressure and also the behavioral response to fear. In their previous work, the authors demonstrated that heart-specific transgenic mIGF-1 mice are able to recover heart function after damage by the modulation of inflammatory response, enhancement of antioxidative cell defenses, and de novo vascularization. This impact of IGF-1 on the mouse heart may be operative through the activation of SIRT1, since in the absence of SIRT1expression in cardiac muscle, mIGF-1 activity could not protect the heart from oxidative stress and lethality [6].

To explain why the cardiomyocytes of mIGF-1 mice respond better to specific types of stress (e.g. ischemia, angiotensin II, cardiotoxin injection, paraquat injection) Bolasco *et al* performed transcriptomic analyses on the hearts of mIGF-1 transgenic versus wild type mice, in absence of any injury. Interestingly, the variations in the mIGF-1/SIRT1 genomic and transcriptomic effects were related to genes implicated in the immune response, the blood pressure control, inflammation and behavior. This suggests that:

1. IGF-1/SIRT1 pathway regulates functions beyond cardiomyocyte specific homeostatic and protective mechanisms,
2. the cardioprotective mIGF-1/SIRT1 pathway might elicit a global impact on body functions.

Of interest was the observation that in absence of damage, local mIGF-1 cardiac expression induces an up-regulation of relevant genes associated with the regulation of immune function. How this “supra-basal” activation of the immune system is compatible with a better cardiac function?

The impact of cardioprotective mIGF-1 pathway on immune and cardiovascular stress is coherent with the concept of hormesis, which is an adaptive response of cells and organisms to a moderate stress. Recent findings have elucidated the cellular signaling pathways and molecular mechanisms that mediate hormetic responses which typically involve enzymes such as kinases and deacetylases, and transcription factors [7]. In this context, SIRT1, an important member of the Sirtuin family of nicotinamide adenine dinucleotide (NAD+)-dependent protein deacetylases, might participate in the hormetic response of mIGF-1 cardiac muscle. The activation of the immune system, by cardiac mIGF-1 expression, would also suggest that mIGF-1 guarantees a constant turnover of cardiac stem cells and cardiomyocytes. Of note, different studies of the group of Rosenthal, Musarò and Vinciguerra clearly indicated that mIGF-1 exerts its anabolic role, in both cardiac and skeletal muscle, acting on stem cell compartments. Another important and surprisingly finding of the study by Bolasco et al. was the observation that mIGF-1 transgenic mice were mildly hypertensive and this effect could be rescued by cardiomyocyte specific depletion of SIRT1. Although the authors did not define a causal link between high blood pressure and cardiac mIGF-1/SIRT1 pathway, it is possible that the activation of immune system plays a critical role in the modulation of blood pressure. Indeed, inflammation is recognized as an important factor in the pathophysiology of hypertension [8]. Although the hypertension is considered a pathologic events which also favors a decline in memory, a study in centenarians showed conversely that high blood pressure is associated to improved cognitive functions [9]. Interestingly, Bolasco and collaborators demonstrated that mice harboring mIGF-1 transgene in the heart had a better memory of the aversive stimulus, for both cue and context fear response, in that they spent a significant augmented time in a frozen position compared to wild type littermates. This was strictly dependent on the presence of SIRT1.

Thus, a primed immune system can constitute an hormetic advantage for responding to a potential cardiac tissue damage. In this context, further studies should better address the causes of hypertension, taking into consideration epigenetic phenomena and environmental factors. This would help to better define whether essential hypertension could be replaced by other terms with precise pathophysiological characteristics. Together, these observations support hypothesis that the mIGF-1/SIRT1 pathway maintains the “youth heart” and renders the cardiac muscle more responsive and resistant to different pathologic stimuli.
